# Highlights in the association of fragmented QRS with myocardial fibrosis

**DOI:** 10.55730/1300-0144.5450

**Published:** 2021-11-16

**Authors:** Mehmet EYÜBOĞLU

**Affiliations:** Department of Cardiology, Faculty of Medicine, Gaziosmanpaşa University, Tokat, Turkey

**To the Editor**,

Fragmented QRS (fQRS) on electrocardiography (ECG) is a predictor of myocardial scar and fibrosis and is significantly associated with adverse outcomes in patients with cardiovascular diseases (CVD) [1]. Recently, Dural et al. [2] reported that the presence of fQRS on ECG was significantly associated with left ventricular hypertrophy (LVH) parameters in patients with acromegaly. This study provides useful information in regard to evaluation of patients with acromegaly. However, the term fQRS includes various types of fragmentation patterns in the original QRS complex that have different clinical importance. In this sense, I would like to address some important points to clarify how to use fQRS as a marker of myocardial damage in clinical practice.

The association of fQRS with LVH in patients without hypertension has been previously reported [3]. Moreover, presence of fQRS on ECG also significantly predicts myocardial fibrosis and subclinical myocardial damage in patients without evidence of LVH and CVD [4–7]. In this sense, fQRS is generally considered to be a sign of early stage myocardial fibrosis before the emergence of manifest CVD. However, there are some important issues that should be considered in the association of fQRS with myocardial fibrosis. In the study by Dural et al. [2], the authors did not report any data regarding the number of leads with fQRS and localization of fQRS on ECG. Number of leads with fQRS and localization of fQRS on ECG seem to be important predictors of extent and severity of myocardial damage. Importantly, increased number of leads with fQRS significantly predicts advanced myocardial damage and adverse outcomes [8]. Also, while fQRS in anterolateral leads seems to be associated with myocardial fibrosis and damage, the association of fQRS in inferior leads with myocardial damage remains unclear and may be regarded as benign variant in patients without CVDs [9–10]. Therefore, to assess the association of fQRS with impaired cardiac structure, number of leads with fQRS and its localization on ECG should be taken into consideration.

In conclusion, presence of fQRS on ECG may be useful in the clinical evaluation of patients with acromegaly. However, some types of QRS fragmentation may not be associated with myocardial damage. Hence, localization of fQRS on ECG and number of leads with fQRS should be considered in order to define the exact association of fQRS with impaired cardiac structure and LVH parameters.
